# Advances in biomarkers and techniques for pancreatic cancer diagnosis

**DOI:** 10.1186/s12935-022-02640-9

**Published:** 2022-06-28

**Authors:** Haotian Wu, Suwen Ou, Hongli Zhang, Rui Huang, Shan Yu, Ming Zhao, Sheng Tai

**Affiliations:** 1 Department of General Surgery, The Second Affiliated Hospital of Harbin Medical University, Harbin, 150086 China; 2 Harbin Medical University, Harbin, 150086 China; 3 Department of Pathology, The Second Affiliated Hospital of Harbin Medical University, Harbin, 150086 China; 4 Department of Gastroenterology, The First Affiliated Hospital of Chengdu Medical College, Sichuan, 610500 China

**Keywords:** Pancreatic cancer, Biomarkers, Artificial intelligence, Nanomaterials, Diagnosis

## Abstract

Pancreatic cancer is the most lethal type of malignancy and is characterized by high invasiveness without severe symptoms. It is difficult to detect PC at an early stage because of the low diagnostic accuracy of existing routine methods, such as abdominal ultrasound, CT, MRI, and endoscopic ultrasound (EUS). Therefore, it is of value to develop new diagnostic techniques for early detection with high accuracy. In this review, we aim to highlight research progress on novel biomarkers, artificial intelligence, and nanomaterial applications on the diagnostic accuracy of pancreatic cancer.

## Introduction

Pancreatic cancer (PC) is the second leading cause of cancer-related deaths in the United States, and the estimated numbers of new cases and deaths are 60,430 and 48,220 in 2021, respectively. The 5-year survival rate is as low as 10%, despite the advent of new drugs in the past decade [1]. The imperceptible symptoms at an early-stage cause patients to miss their best chance for diagnosis and therapy. It is generally accepted that the combination of surgical resection with postoperative chemotherapy serves as the most effective strategy for PC treatment. Surgical resection alone may provide a cure for PC at an early stage, especially for tumors with a diameter less than 1 cm. Moreover, surgical resection achieved a 5-year survival rate of 80.4% for PC patients whose tumors were 1 cm or less. When PC patients have UICC stage 0, stage I A, and stage I B disease, the postoperative 5-year survival rates are 85.8%, 68.7%, and 59.7%, respectively [2].

### Traditional diagnostic methods

At present, there remain challenges in developing imaging examination and tumor markers for early-stage PC diagnosis. The imaging diagnostic methods include abdominal ultrasound, CT, MRI, and endoscopic ultrasound (EUS) [3]. Ultrasound examination sometimes has difficulty accurately capturing small PC lesions due to the interference of gases in the gastrointestinal tract, preventing it from being a screening method for early-stage PC detection [4]. Enhanced CT is the first choice for the diagnosis of early-stage PC, with an overall sensitivity between 76 and 92%. However, if PC tumors less than 2 cm in diameter were included in the CT detection, the sensitivity dropped to 63–77% [5]. MRI can complement CT in the diagnosis of PC with sensitivity and specificity both at 89% [6, 7]. The detection rates of EUS for PC in stage 0 and stage I were 45.5% and 81.8%, respectively, compared with 9.7% and 63% for CT and 9.7% and 39.1% for MRI [8]. As an invasive examination causing wounds, EUS still requires improvement for the accuracy of early-stage PC diagnosis. In addition, the diagnostic accuracy is affected by the subjectivity of different clinicians. Therefore, there is an urgent need to develop novel diagnostic agents and methods. Our review highlights the research progress of novel biomarkers, artificial intelligence technology, and new nanomaterials in the diagnosis of early-stage PC which can be resected more easily.

## Advance of biomarkers related to pancreatic cancer

Serum molecules, including CA19-9, CA125, and CEA, are widely used tumor markers for routine PC detection. Serum marker detection is superior to the abovementioned traditional methods in terms of high reproducibility, good patient compliance, easy follow-up, and low cost. According to previous research, the median sensitivity and specificity of CA19-9 are 79% and 82%, respectively [9]. However, CA19-9 is not tumor type-specific because of its elevation in other malignancies, including colorectal cancer, cholangiocarcinoma, hepatocarcinoma, gastric cancer, and even benign diseases, such as obstructive jaundice, cirrhosis, cholangitis, and other gastrointestinal diseases [10]. Notably, a high CA19-9 level usually suggests advanced PC instead of early-stage PC, especially for tumors with a diameter less than 3 cm [11, 12]. Of note, the combination of CA19-9, CEA, CA125, and CA242 showed high sensitivity and specificity for PC diagnosis, with up to 90.4% and 93.8%, respectively, which are significantly higher than the accuracy of a single serum marker [13].

### Molecular biomarkers

#### DNA methylation

It is believed that DNA methylation plays an important role in the initiation and progression of PC by rebalancing tumor suppressor genes and proto-oncogenes [14–16]. The detection of DNA methylation in peripheral blood is prevalent for PC diagnosis. Promoter DNA methylation of ADAMTS1 and BNC1 was significantly associated with PC in a cohort of 123 patients with PC, 20 patients with pancreatic intraepithelial neoplasia and 30 patients with pancreatitis, with sensitivities of 79% and 48% and specificities as high as 92% and 89%, respectively. Additionally, the sensitivity and specificity of the two-marker combination for PC diagnosis are 81% and 85%, respectively [17]. Moreover, the DNA methylation of ADAMTS1 and BNC1 was reported to be an ideal biomarker for PC TNM stage estimation. In regard to stage I PC patients, the percentages of ADAMTS1 and BNC1 DNA methylation were 87.5% and 62.5%, respectively; for stage IIa, the percentages were 77.8% and 55.6%, respectively; for stage IIb, the percentages were 90% and 65%, respectively; and for late-stage (III + IV), the percentages were almost 100%. When the two markers were used together, the percentages were 100%, 88.9%, 100%, and 100% in patients at stages I, IIA, IIB, and III/IV, respectively, suggesting an inspiring translational future [18].

Another study in Japan revealed that DNA methylation of CDO1 was related to the early diagnosis of PC based on pancreatic cytology specimens from 37 patients with PC and 6 patients with benign pancreatic disease (4 chronic pancreatitis and 2 autoimmune pancreatitis). The results showed that CDO1 promoter methylation was detected in 35/37 (94.6%) PC patients with methylation values (MV) higher than 5.0, while it was not detected in benign diseases (statistically significant, AUC = 0.96, 0.0001) [19]. Furthermore, a case–control study on CD1D DNA methylation was performed in 61 PC patients (stage I, II), 22 patients with chronic pancreatitis, and 19 healthy people with pancreatic juice specimens. Compared with healthy people and chronic pancreatitis patients, the AUC value of CD1D methylation in the pancreatic juice of PC patients was 0.92, with a sensitivity of 75% and a specificity of 95% [20]. The DNA methylation of biomarkers for PC detection is displayed in Table 1.

**Table 1:** DNA methylation of biomarkers for PC detection

DNA methylation of biomarkers	Diagnostic power		
	Sensitivity	Specificity	AUC
ADAMTS1	79%	92%	
BNC1	48%	89%	
ADAMTS1 + BNC1	81%	85%	
CDO1	–	–	0.96
CD1D	75%	95%	0.92

#### Noncoding RNAs

##### MicroRNAs

Noncoding RNAs play important roles in PC development. In a study of blood samples from 409 PC patients, 25 chronic pancreatitis patients, and 312 healthy people, ectopic expression of microRNAs was found in the serum of PC compared with chronic pancreatitis and healthy people. Compared with the control, 38 microRNAs were detected with ectopic expression in early-stage PC patients, and 14 were used to set up two panels for diagnosis (Panel I: miR-145, miR-150, miR-223, miR-636; Panel II: miR-26b, miR-34a, miR-122, miR-126, miR-145, miR-150, miR-223, miR-505, miR-636, miR-885-5p). The two panels were applied to a validation cohort including 180 cases of PC, 1 patient with chronic pancreatitis, and 199 healthy controls. The AUC of Panel I was 0.86, and the diagnostic sensitivity was 85% with a specificity of 64%. Panel II achieved an AUC of 0.93, diagnostic sensitivity of 85% and specificity of 85%. In addition, the panel combined with CA19-9 could further improve the diagnostic efficiency. The AUC of Panel I plus CA19-9 increased to 0.94 (P = 0.01), and Panel II could be up to 0.93 [21]. Except for the above panels, there were many miRNAs that were demonstrated to contribute to the diagnosis of early-stage PC, such as a panel containing 6‐miRNAs (let‐7b‐5p, miR‐192‐5p, miR‐19a‐3p, miR‐19b‐3p, miR‐223‐3p and miR‐25-3p), serum miR-25 combined with CA19-9, and miR-17-5p methylation, which was superior to CA19-9 or CEA [22–24]. Except for pancreatic tissue and blood, miRNA dysregulation was also detected in feces, urine, and saliva, which are easy to obtain by noninvasive methods [25]. In urine, the levels of miR-143, miR-223 and miR-30 were higher at stage I, and the combination of miR-143 and miR-30 showed high sensitivity and specificity with 83.3% and 96.2%, respectively [26]. In stool, Yang et al. reported that the levels of miR-21 and miR-155 were much higher in PC patients than in healthy controls [27]. Recently, salivary miRNAs were demonstrated to be stable due to the protection of exosomes or protein complexes, thus showing their promising roles as diagnostic markers. For example, miR-1246 and miR-4644 in saliva had ROC curves with AUC = 0.814 (P = 0.008) and 0.763 (P = 0.026), respectively, for distinguishing PC patients from healthy controls. Additionally, their combination increased the AUC to 0.833 (P = 0.005) [28]. The efficacy of microRNAs in the differential diagnosis of PC from healthy participants is displayed in Table 2.

**Table 2:** Efficacy of microRNAs in the differential diagnosis of pancreatic cancer from healthy participants

Study	MicroRNAs	source	Diagnostic power							
			AUC		Sensitivity		Specificity		Accuracy	
			Training	Validation	Training	Validation	Training	Validation	Training	Validation
Schultz et al	Panel I	Blood	0.86	0.83	0.85	0.85	0.64	0.48	0.74	0.72
	Panel I + C*	Blood	0.93	0.94	0.85	0.85	0.95	0.98	0.90	0.89
	Panel II	Blood	0.93	0.82	0.85	0.85	0.85	0.55	0.85	0.75
	Panel II + C*	Blood	0.97	0.93	0.85	0.85	0.98	0.90	0.92	0.86
Zou et al	Six‐miRNA panel	Serum	0.910	0.978	0.953	0.933	0.767	0.96		
Yu et al	miR-25	Serum		0.939		0.825		0.9364		0.8895
	miR-25 + C*	Serum		0.985		0.975		0.9011		0.9895
Debernardi et al	miR-143	Urine		0.862		0.833		0.885		
	miR-143 + miR-30	Urine		0.923		0.833		0.962		
Yang et al	miR-21 + miR-155	Stool		0.8111		0.9333		0.6667		
	miR-21 + miR-155 + miR-216	Stool		0.8667		0.8333		0.8333		
Machida et al	miR-1246	Salivary		0.814		0.667		1.0		
	miR-4644	Salivary		0.763		0.750		0.769		
	miR-1246 + 4644	Salivary		0.833		0.833		0.923		

C* = CA19-9. Panel I is composed of miR-145, miR-150, miR-223, miR-636; Panel II is composed of miR-26b, miR-34a, miR-122, miR-126, miR-145, miR-150, miR-223, miR-505, miR-636, miR-885-5p; six‐miRNA panel contains let‐7b‐5p, miR‐192‐5p, miR‐19a‐3p, miR‐19b‐3p, miR‐223‐3p, and miR‐253p

##### LncRNAs

In addition to microRNAs, long noncoding RNAs may also serve as effective biomarkers for early-stage PC detection. For example, compared with healthy controls, the SNHG15 level is higher in PC patients and contributes to cell proliferation via H3K27me3 mediated by EZH2 [29]. In addition, SNHG15 expression in serum exerts a moderate diagnostic value with a sensitivity of 68.3% and a specificity of 89.6% [30]. Plasma lncRNA Linc-pint was significantly decreased in PC patients compared with healthy volunteers, as well as in carcinoma of the ampulla of Vater and cholangiocarcinoma. Therefore, Linc-pint might be used for identifying the cause of malignant obstructive jaundice and helping to trace the cancer origin [31].

##### CircRNAs

Circular RNAs (circRNAs) have continuous closed circular structures, making them stable enough to serve as molecules for cancer detection [32]. Circ-LDLRAD3 was significantly increased in PC tissues and plasma, and markedly related to lymphatic invasion, venous invasion, and metastasis. Although circ-LDLRAD3 is not an ideal independent biomarker, its combination with CA19-9 showed an increase in AUC from 0.87 to 0.67, and the sensitivity and specificity were 80.33% and 93.55%, respectively [33]. The circRNAs IARS and PDE8A that are contained in the plasma exosome were upregulated and associated with the progression and prognosis of PC, and are likely to be promising biomarkers in the detection of early-stage PC [34, 35]. In addition, compared with the healthy controls, circ-001569 levels were higher in 26 tumor tissues and 97 plasma samples of PC patients (P < 0.01) [36]. Table 3 shows the efficacy of lncRNAs or circRNAs in the differential diagnosis of PC from healthy participants.

**Table 3:** Efficacy of lncRNAs or circRNAs in the differential diagnosis of pancreatic cancer from healthy participants

Study	Markers	source	Diagnostic power		
			AUC	Sensitivity (%)	Specificity (%)
*lncRNAs*					
Guo et al	SNHG15	Serum	0.727	68.3	89.6
Li et al	Linc-pint	Plasma	0.87	87.5	77.1
	Linc-pint + CA19-9	Plasma	0.92	85.9	82.9
*circRNAs*					
Yang et al	circ-LDLRAD3 + CA19-9	plasma	0.87	80.33	93.55

#### Proteomic biomarkers

Proteomics is based on the study of the full set of proteins and aims to understand all expressed proteins in cells, including their number, level, and renewal. Protein biomarkers related to PC can be detected in the patient’s blood, pancreatic juice, and tumor tissue [37].

Aberrant levels of GPC1, CPA4, C4BPA, PFAA, MUC5AC, and OPNT + TIMP-1 were frequently detected in the serum of PC patients. Melo’s team found that the level of GPC1 in exosomes from the blood of PC patients was significantly higher than that in exosomes from patients with benign pancreatic diseases and healthy people. In addition, the GPC1 expression level is positively correlated with the tumor burden [38]. Similarly, Sun et al. affirmed the potential of CPA4 as a great biomarker of PC. They compared the serum levels between PC patients (n = 100) and healthy patients (n = 50). The results suggested that PC patients had significantly greater serum levels of CPA4 than patients in the healthy group (1.695 ± 2.093 vs. 0.123 ± 0.251 ng/mL, P = 0.000) [39]. Notably, C4BPA is superior to CA19-9 in sensitivity and specificity in the early diagnosis of PC [40]. PFAA also has a strong correlation with the stage of PC and could be used as a pathological diagnostic reference [41]. Sukhwinder et al. supported MUC5AC as a valuable biomarker for PC detection. MUC5AC exhibited satisfactory sensitivity and specificity when used in the differential diagnosis among PC, benign pancreatic disease, and chronic pancreatitis. A similar function was also shown when MUC5AC was combined with CA19-9 [42]. Serum osteopontin and tissue inhibitor of metalloproteinase 1 (OPNT + TIMP-1) combined with CA19-9 in blood also displayed potential to improve the sensitivity of PC diagnosis [43]. In regard to the protein markers in urine samples, the levels of LYVE1, REGIA, and TFFI were significantly related to PC. The accuracy exceeds 90% for the early diagnosis of PC; thus, it showed an ideal clinical impact, although it still requires a large cohort for validation [44]. In addition, neutrophil gelatinase-associated lipocalin (NGAL) in urine provides a clue for the early diagnosis of PC [45].

**Fig. 1:**
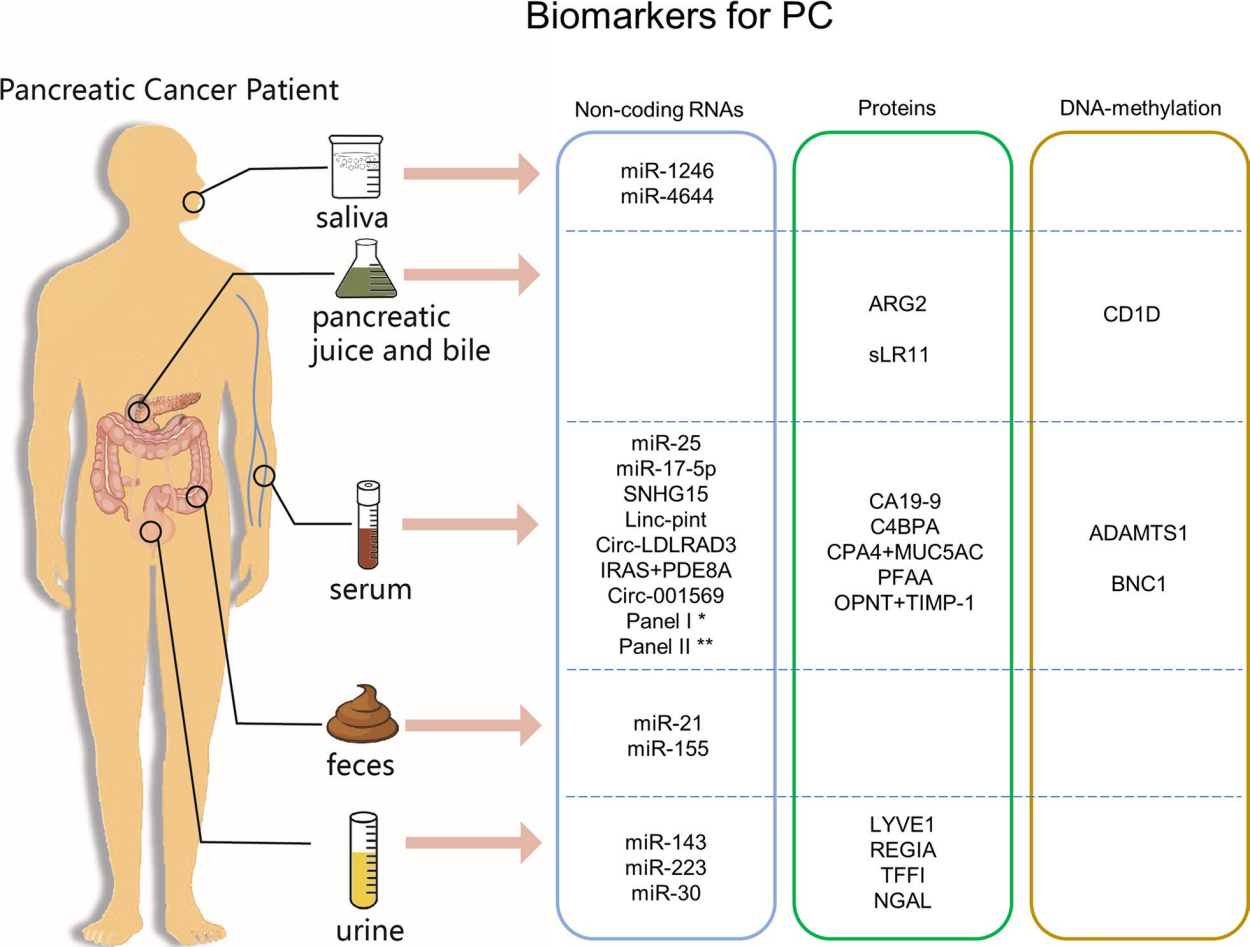
Biomarker candidates for the diagnosis of pancreatic cancer from easy-to-obtain samples in clinics, including saliva, pancreatic juice and bile, serum, feces, and urine. *Panel I = miR-145, miR-150, miR-223, miR-636 **Panel II = miR-26b, miR-34a, miR-122, miR-126, miR-145, miR-150, miR-223, miR-505, miR-636, miR-885-5p

In pancreatic juice, the upregulation of anterior gradient-2 (ARG2) implied its role as a marker for PC diagnosis [46]. However, the acquisition of pancreatic juice requires an invasive method, which is not widely accepted. Moreover, the bile component could be used to detect early-stage PC with high sensitivity [47]. It was reported that LDL receptor-related with 11 ligand-binding repeats (sLR11) in the bile of PC patients has the potential to distinguish PC from healthy controls [48]. All of the above biomarkers from different clinics for PC diagnosis are summarized in Fig. 1.

## Artificial intelligence (AI)

The diagnosis of PC by traditional imaging methods and pathological slices requires experts to perform complex analyses on a large amount of data. Due to differences in physician training, experience, and professional qualities, the results of diagnosis are partly influenced by the subjectivity of doctors. Therefore, it is crucial to develop an automatic and accurate imaging processing technology that requires less manual intervention [49]. In recent years, AI techniques have revolutionized the medical field. With the help of AI technology, tedious image analysis, subjective differences of doctors, and inconsistent diagnosis can be avoided [50]. AI technology is capable of accelerating image processing while maintaining consistency and high accuracy.

The conceptual category of AI primarily includes machine learning (ML) and deep learning (DL) [51]. ML is a multifield interdisciplinary subject involving subjects such as probability theory and statistics. ML specializes in the study of how computers simulate or implement human learning behaviors to acquire new knowledge or skills and reorganize the existing knowledge structure to continuously improve its performance. DL learns the internal laws and representation levels of sample data. These learning processes are very helpful for the interpretation of many texts, images, and sounds. DL is also composed of a variety of deep neural networks, with tremendous reports on its application in the recognition of lung cancer, prostate cancer, and rectal lymph node images with satisfactory results [52–54]. Another successful model is the deep convolution neural network (DCNN), which has been shown to improve the diagnostic precision of thyroid cancer by analyzing clinical ultrasound sonographic imaging results. Compared to a group of professional radiologists, the model showed comparable sensitivity and increased specificity concerning the detection of patients with thyroid cancer [55]. At present, AI has been applied in the field of imaging intelligent diagnosis of pancreatic diseases with significant progress [56, 57].

### AI utilization in PC diagnosis

#### AI utilization in imaging

For CT and MRI, AI has been used to facilitate diagnostic accuracy [58, 59]. The computer learning based on a convolutional neural network (CNN) from 370 PC patients and 320 non-PC patients showed a more superior diagnostic efficiency than radiologists, with an accuracy of 0.986–0.989. The sensitivity of CNN was also higher than that of radiologists (0.983 vs. 0.929; p = 0.014). However, during the test, the CNN technique missed 3 tumors with a diameter < 15 mm, 2 of which were discovered by radiologists. The radiologist missed 12 tumors with a diameter ranging from 10 to 33 mm, while the CNN accurately diagnosed 11 of them. Liu et al. used the CNN method to interpret contrast-enhanced CT images of 338 PC patients (238 as the training set with 4385 images and 200 patients with 1699 images as the validation group) [60]. The R-CNN model was used to classify the recognized images. The true-positive rate (TPR), false-positive rate (FPR), precision (P), and recall rate (R) were obtained according to whether the sample was accurately identified and the coverage rate of the identification. Subsequently, the average value of average precision (AP) for each group was obtained. The mean AP of the R-CNN for 4385 image recognition for training was 0.7664. The receiver operating characteristic (ROC) curve for the validation group showed the area under the curve (AUC) according to the trapezoidal rule to be 0.9632. These results highlight the effectiveness of AI in the auxiliary diagnosis of PC. The team also noticed that AI only took 0.2 s to recognize one CT image and 3 s to finish the image recognition of one patient, which is much faster than the average recognition time of a well-trained imaging doctor (average 8 min) [61]. Furthermore, Gao et al. also used the CNN model to identify MRI images with a 0.90 AUC of PC [62]. The combination of PET/CT images with AI technology used the support vector machine-random forest (SVM-RF) + dual-threshold principal component analysis (DT-PCA) model to detect PC. The sensitivity/specificity/accuracy was as high as 95.2%/97.5%/96.5%, suggesting that the diagnostic efficiency of PET-CT was significantly improved with the assistance of AI [63].

The major difficulty for EUS-AI applications is that different lesions often have similar imaging findings. The subjectivity of the surgeon is also a factor, causing a certain proportion of misdiagnoses and missed diagnoses. Based on the principle of pixels, AI can reduce subjectivity by integrating the changes in the lesion structure with digital image analysis. In a controlled experiment from Das et al., they established an artificial neural network (ANN) based on different pathological types of PC, chronic pancreatitis, and normal pancreas. The ANN model displayed a high sensitivity/specificity/AUC of 93.0%/92.0%/93% for PC detection, as well as a perfect discrimination of normal pancreas and chronic pancreatitis (sensitivity, specificity, and accuracy are as high as 100%) [64]. Zhu et al. conducted a comparative analysis of 262 cases of PC and 126 cases of chronic pancreatitis, including the support vector machine model’s recognition of 16 EUS images for each patient. The sensitivity/specificity/accuracy of the model is up to 91.6%/95.0%/94.2% for PC detection [65]. The ANN model for the enhanced EUS images in another study showed that vascular parameters can distinguish PC and chronic pancreatitis cases with a sensitivity of 94.64% and a specificity of 94.44% [66].

#### AI utilization in pathology

AI attempts to use computer models for pancreatic cytological diagnosis, especially on samples from endoscopic ultrasonography-guided fine needle aspiration (EUS-FNA). The images of cell cluster fragments under pathological slices were obtained under the analysis from a mobile neural network (MNN) model to distinguish benign and malignant cells with features [67]. These features are surprisingly similar to those recognized by cytopathologists. This suggests that MNN have comparable accuracy with pathologists in their preliminary judgments on cell images. The study also showed an MNN model of 80% for the sensitivity of the FNA results that the pathologist could not determine. Collectively, these findings indicate the MNN model is a promising tool for pancreatic FNA specimen screening and reducing the limitations of pathologists’ subjective judgment.

#### AI utilization in biomarkers

AI can also be applied for the analysis of biological markers [68]. In a study with AI to analyze serum tumor markers (CA19-9, CA125, CEA), the 913 serum samples from PC patients and non-PC patients were randomly divided into a training group (sample size of 658) and a test group (sample size of 255). They established an ANN model for PC diagnosis in the training group and validated the model in the test group. The results showed that the AUC of PC was as high as 0.91. Compared with the AUC (CA19-9: 0.845, CA125: 0.795, CEA: 0.800) of a single tumor marker obtained under the Logistic Regression model, the ANN model displayed a better diagnostic performance [69]. Researchers used a smoothly clipped absolute deviation-based penalized support vector machine to build a PC diagnosis model in another study, with 39 miRNA markers in blood serum samples from 63 PC patients and 63 control subjects as the training cohort, and it was validated in an additional group with 25 PC samples and 81 intrahepatic cholangiocarcinoma samples. The AUC, sensitivity (96.0%), and specificity (90.0%) of the proposed diagnostic model were 1.5, 1.3, and 2 times higher than those of the CA19-9 diagnosis model, respectively [70]. Although the individual miRNAs were not specific to PC, the combination of all 39 miRNA markers enabled a high diagnostic specificity. The diagnostic power of the individual models is summarized in Table 4.

**Table 4:** Applications of AI in the diagnosis of pancreatic cancer

Study	Diagnostic methods	AI emulator	Diagnostic power			
			Accuracy (%)	Sensitivity (%)	Specificity (%)	AUC
Liu et al	CT	CNN				0.96
Gao et al	MRI	CNN				0.90
Das et al	EUS	ANN	93.0	92.0		0.93
Yang et al	Biomarkers	ANN				0.91
Li et al	PET-CT	SVF-RF + DT-PCA	95.2	97.5	96.5	
Zhu et al	EUS	SVM	91.6	95.0	94.2	
Momeni et al	EUS-FNA	MNN		80		

## Application of nanomaterials in the diagnosis of pancreatic cancer

Novel nanomaterial components are believed to act as a powerful tool for improving the sensitivity and specificity of early-stage cancer detection [71–74].

### Nanomaterials as contrast agents

The addition of novel designed nanoparticles (NPs) to contrast agents could overcome the limitations of first-generation organic contrast agents by increasing sensitivity through better biodistribution [75]. In 2015, Rosenberger et al. modified the nanoparticle of a peptide with a high affinity to galectin-1, which is highly expressed in PC cells, thus making it an ideal MRI contrast agent [76]. Luo et al. also reported a new type of nanocontrast agent—iron oxide NPs (IONPs), which were encapsulated in HA-Fe_3_O_4_-NPs (hyaluronic acid-mediated multifunctional Fe_3_O_4_ nanoparticles) with fluorescein isothiocyanate (FITC) together to capture the NP by using the principle of selective recognition of hyaluronan by CD44 receptor [77]. In both cases, the accumulation of NP in the tumor increased the detection accuracy by MRI. In terms of safety, IONPs are superior to the current organic contrast agents used in toxic reduction [78].

Another nanomaterial contrast agent named AuNR–SiO_2_–GD NPs was injected into PC-bearing albino mice followed by CT and MRI examinations, showing an increased contrast effect, higher detection sensitivity, more accurate target biodistribution, and more accurate spatial and temporal resolution. In terms of safety, the contrast agent accumulated in the liver without damage [79–81].

### Nanomaterials as sensitizers of biomarkers

Another team tried carbon nanotubes (CNTs) carrying CA19-9 antibody and performed detection experiments on CA19-9 at different concentrations. The results showed that the CNT-based detection threshold was 100 times lower than that of the traditional ELISA method. Therefore, it is possible that CNTs may detect changes in CA19-9 levels at the early stage of PC [82]. Multiwalled carbon nanotubes (MWCNTs) developed by another lab were used as a sensitive biosensor carrier to detect low levels of CA19-9 [83]. The specific antibody attached to the surface of MWCNTs on the test paper was able to detect CA19-9 at a concentration of 0–1000 U/mL in the blood sample.

Quantum dots (QDs) are also a type of nanoparticle used to detect cancer. The fluorescence excitation wavelengths of QDs range from 400 to 2000 nm, and the size and composition of QDs can be adjusted for application. This characteristic makes it possible for a single light source to detect and track multiple biomarkers at the same time [84]. Furthermore, QDs are reusable and have a longer line span due to their fading resistance [85]. ZnO QDs, as electrochemical and fluorescent labels, are used for the detection of the PC biomarker CA19-9 by an immunosandwich method with high sensitivity, selectivity, and stability. Based on the bioconjugation of ZnO QDs and the CA19-9 antigen-antibody immunoreaction, sandwich immunosensors were assembled on a functional Si substrate for CA19-9 detection. Immune recognition of CA19-9 was converted into amplified signals of square wave stripping voltammetry and intrinsic photoluminescence. In this way, the peak intensity of square wave voltammetry rose with an increase in CA 19-9 concentrations, showing a broad linear response range from 0.1 to 180 U/mL in logarithmic styles [86]. The detection limit of 0.1 U/mL CA 19-9 is far less than the threshold concentration in clinical diagnosis value of 35 U/mL, suggesting the power in the diagnosis of PC at an early stage.

### Nanomaterials as diagnostic probes for PC

The monodispersed organically modified space silica (ORMOSIL) nanoparticles, which were covalently conjugated with the fluorophore rhodamine, presented a variety of active groups on their surface and were used to detect early-stage PC. The carboxyl groups on the surface were conjugated to bioactive molecules, such as monoclonal antibodies, to target unique antigen molecules on PC cells. ORMOSIL nanoparticles entered PC cells in a receptor-mediated manner. Detection of the conjugated fluorophore rhodamine showed that the uptake of nanoparticles conjugated with anti-claudin 4, anti-mesothelin, and transferrin was much higher in PC cells than in control cells (more than 90% vs. 55.9%). In addition, ORMOSIL selectively targeted tumor cells and did not show any cytotoxicity in vitro [87]. Therefore, the property of the fluorescent ORMOSIL nanoparticles showed potential applications for optical bioimaging as effective probes for diagnosis in vivo. The functions of various nanomaterials are summarized in Table 5

**Table 5:** The function of nanomaterials for PC detection

Study	Nanomaterials	Fuction
Rosenberger et al	Nanoparticle of a peptide with a high affinity to galectin-1	Contrast agents
Luo et al	IONPs	Contrast agents
Boyer et al	AuNR–SiO_2_–GDNPs	Contrast agents
Zhuo et al	CNTs	Sensitizers of biomarkers
Jin et al	MWCNTs	Sensitizers of biomarkers
Gu et al	ZnO QDs	Sensitizers of biomarkers
Kumar et al	ORMOSIL	Diagnositic probes

.

## Future perspective

There is a debate about finding a sensitive biomarker to replace CA19-9 for predicting early-stage PC. Emerging novel biomarkers and AI techniques in imaging have enabled the precision diagnosis of early-stage PC in recent decades. As genetic changes are prevalent in the initiation and progression of PC, molecular biomarkers, including DNA methylation, noncoding RNAs, and proteins from peripheral blood or pancreatic juice, exerted their roles as indicators in PC detection. However, there remains many limitations for clinical translation due to the inconsistence of sensitivity and specificity. DNA methylation seems to have a very stable detection rate, but methylation did not have a very strong relationship with early-stage PC. The dysregulation of noncoding RNAs was intensively investigated and detected in blood, feces, urine, or saliva, which were more acceptable by the patients, compared with other invasive detection methods, such as EUS and FNA. Moreover, circRNAs showed great stability in body fluids, making them ideal biomarkers for PC diagnosis. To further enhance the sensitivity and specificity of early detection, an effective cocktail combination model should be developed before clinical translational validation.

AI is mainly used for the recognition of images of CT, MRI, EUS, and pathology examinations. The identification accuracy of AI depends on not only the construction and optimization of the neural network model but also the size of the dataset used for training. AI already helps pathologists overcome tedious image analysis, eliminate subjective differences, and enhance diagnosis consistency. In many cases, AI performance is superior to pathologists in cytomorphology. In this aspect, AI can be used to judge pancreatic FNA specimens or cells in pancreatic juice that pathologists cannot determine and provide a reference value for the early diagnosis of the disease, improving early intervention for PC.

Nanomaterials have attracted much attention in the field of medical diagnosis as contrast agents for imaging examination with a high affinity for PC cells due to their conjugated active molecules on the surface. In addition, nanomaterials have better biodistribution, contrast enhancement, and safety in the human body than traditional contrast agents. Nanomaterials can also be modified with antibodies, which can bind specifically to biomarkers on cells. The PC serum biomarker detection system combined with nanomaterials has a much wider range of detection and a lower detection threshold, which is probably significant in the diagnosis of early-stage PC. Nanoparticles conjugated with fluorescence could help in the optical bioimaging of PC, but the intensity remains a problem. Importantly, most nanomaterials are in the laboratory research stage and have not been tested in trials. There is still a long way before clinical application of nanomaterials because of their potential toxicity, side effects, and efficacy in humans.

At present, these new diagnostic methods also have great potential to be put into clinical practice. The new biomarkers detecting PC depend on the technology of fluid biopsy, which can perform a non-invasive examination in peripheral blood or exocrine fluids. The critical step in putting these new biomarkers into clinical practice is developing efficient and sensitive test kits. In the past, the gold standard for testing DNA methylation was bisulfite sequencing. However, this method also had the defects of low accuracy and poor repeatability, while a kind of digital PCR (dPCR) technology currently under research could overcome the above limitations, and the kits based on this technology have good clinical prospects. Meanwhile, simple and sensitive single-molecule fluorescence technology has made great progress in detecting noncoding RNAs. However, it is limited to detecting the nucleic acids from extracellular fluid, but some relevant nanosensors can detect noncoding RNAs in the living cells. It is possible to attain the clinical practice of testing noncoding RNAs by the above two modes. Moreover, the routine immunohistochemical technology could be used in the detection of novel proteomic biomarkers according to the combination of antigens and antibodies in peripheral blood, saliva, urine, feces and pancreatic juice. Increasing the volume of collected specimens, and developing concentration technology may be a solution to developing applicable clinical apparatus based on the multiple characteristics of new biomarkers. In addition, the digital pathology with AI technology has made great progress, which can realize a series of functions such as image collecting, image preprocessing, image segmentation, feature acquisition, image classification and recognition. The pathological and cytological robots based on the above techniques have been well applied to the cytological examination and the rapid freezing pathological detection to improve the accuracy and speed of diagnosis. Significantly, the application of AI technology does not mean that it can completely replace the roles of medical practitioners in the judgment of diseases, which is only used as a reference in clinical practice. At the same time, an AI corrective system for diagnosis should also be established to check the conclusion of clinicians in time and improve the accuracy of diagnosis. Furthermore, when the nanomaterials are used as contrast agents in the imaging examination of PC, it is feasible to improve the safety by means of controlling the size of nanomaterials before clinical practice. Using biocompatible molecules such as folic acid or glucose to modify the nanomaterials may also improve the compatibility of human tissue or increase the binding affinity to cancer cells. The diagnostic probes which are rich in nanomaterials can play their optical characteristics to locate the PC lesions or conduct immunofluorescence labeling for final clinical practice.

**Fig. 2:**
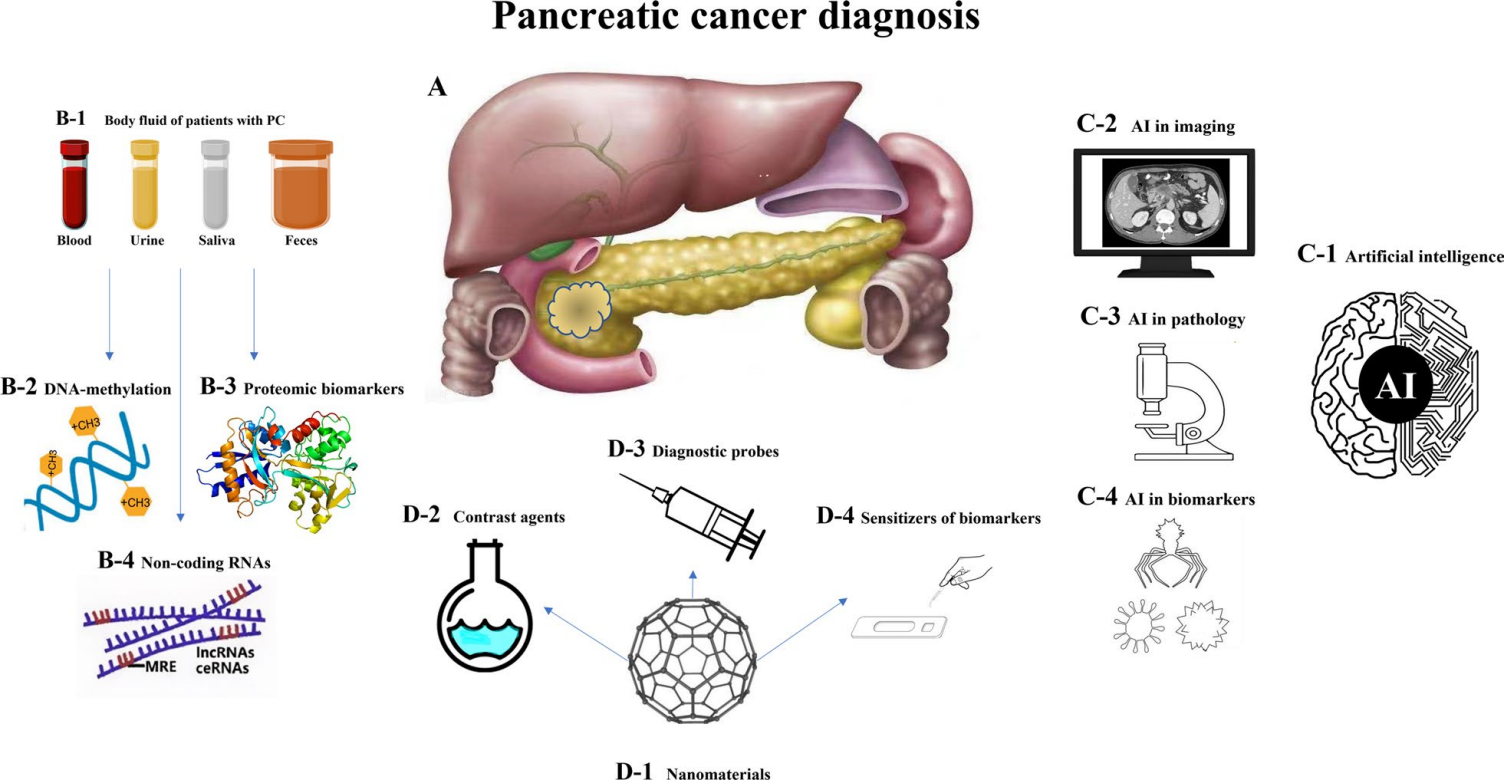
Potential clinical applications for the diagnosis of **A** pancreatic cancer, including **B** new biomarkers, **C** Artificial intelligence (AI), and **D** nanomaterias. As shown in Part **B**, samples were obtained from urine, blood, saliva, and feces such as **B-1**; new biomarkers included DNA methylation such as **B-2**, proteomic biomarkers such as **B-3**, and noncoding RNAs such as **B-4**. AI is shown in Part **C**. AI utilization can be used in imaging such as **C-2**, pathology such as **C-3** and recognition of biomarkers such as **C-4** for PC detection. Part of **D** describes the application of nanomaterials for the diagnosis of PC; they can play a role as contrast agents such as **D-2**, diagnostic probes such as **D-3**, and sensitizers of biomarkers such as **D-4**

The question of how to diagnose PC earlier is always a motivation for researchers. Screens for early-stage PC need more sensitive and tumor-type specific biomarkers. The ideal method of biomarker detection should be less affected for patients and simple to conduct by clinicians. With the advancement of algorithms and neural network models, AI-assisted PC diagnosis will be used widely in clinical practice. The ultimate goal of AI techniques is to allow machines to have the ability to analyze and learn like humans and make diagnostic decisions for clinicians. The improvement of current methods and the development of novel concepts for diagnosis are necessary for the near future (Fig. 2).

With the progress of research, we believe that imaging examinations, biomarker tests and other novel methods for early-stage PC diagnosis would benefit the PC patient survival rate.

## Data Availability

Not applicable.

## Abbreviations

AI: Artificial intelligence
AP: Average precision
ANN: Artificial neural network
AUC: Area under the curve
CEA: Carcinoembryonic antigen
CT: Computed tomography
CNTs: Carbon nanotubes
CNN: Convolutional neural network
DL: Deep learning
DCNN: Deep convolution neural network
DT-PCA: Dual-threshold principal component analysis
EUS-FNA: Endoscopic ultrasonography-guided fine needle aspiration
FPR: False-positive rate
MRI: Magnetic resonance imaging
EUS: Endoscopic ultrasound
PC: Pancreatic cancer
ROC: Receiver operating characteristic
SVM-RF: Support vector machine-random forest
TPR: True-positive rate
NP: Nanoparticle
MV: Methylation values
ML: Machine learning
MNN: Mobile neural network
NGAL: Neutrophil gelatinase-associated lipocalin
